# Further psychometric evaluation of the Swedish version of the Interdisciplinary Education Perception Scale (IEPS) using Rasch analysis

**DOI:** 10.1186/s12909-025-08553-1

**Published:** 2026-01-17

**Authors:** Sara von Wallenberg Pachaly, Caisa Öster, Mia Ramklint, Mathilde Hedlund Lindberg

**Affiliations:** 1 Department of Medical Sciences, Child and Adolescent Psychiatry, Uppsala University, Uppsala, Sweden; 2 Department of Medical Sciences, Psychiatry, Uppsala University, Uppsala, Sweden; 3 Psychiatric Services Lucerne, Lucerne, Switzerland

**Keywords:** Interdisciplinary Education Perception Scale (IEPS), Psychometric properties, Rasch analysis

## Abstract

**Background:**

The Interdisciplinary Education Perception Scale (IEPS) is an instrument that measures students’ perceptions of interprofessional learning. It has shown satisfactory internal consistency for the total scale; however, problems regarding the psychometric properties of the subscales have resulted in several different versions of the instrument. The aim of this study is to further evaluate the psychometric properties of the Swedish version of IEPS using Rasch analysis.

**Methods:**

The Swedish translation of the 12-item, 3 subscale version of the IEPS was used in a student population (*n* = 704) from eight healthcare programmes. The psychometric properties were evaluated using Rasch analysis based on five aspects: rating scale functioning, unidimensionality, reliability, targeting, and differential item functioning.

**Results:**

The total scale showed satisfactory internal consistency. However, the scale was suboptimal regarding the response categories, where three response categories had to be combined into one to achieve a more optimal number of observations per category. Regarding the second subscale, *Perceived need for cooperation,* item 4 and particularly item 6 showed local dependency and disordered thresholds.

**Conclusion:**

To improve the evaluation of interprofessional education, future research should rephrase the response categories or minimize the number of response options. Additionally, the removal of item 6 and possibly item 4 from the second subscale may improve the psychometric properties of the scale.

## Background

Interprofessional teamwork has been shown to be critical for the provision of effective and efficient health care [18]. Interprofessional competency can be both taught and learnt. Interprofessional education (IPE) is defined as education where students from different programmes learn about, and together with, each other; the learning process is called interprofessional learning (IPL) [25]. IPE aims at providing healthcare students with opportunities to develop their professional roles and their understanding of other professions, as well as to develop their teamwork and communication skills [11]. Since the effects of IPE can be difficult to quantify, previous research has focused on students’ attitudes towards it [17].

The Interdisciplinary Education Perception Scale** (**IEPS) was developed to measure the professionally oriented perceptions of participants in interdisciplinary education programmes [14]. The original scale was developed in a population (*n* = 143) from various healthcare disciplines and consists of 18 items on a 6-point Likert self-report scale: 1 (strongly disagree) to 6 (strongly agree), and has four subscales: *Competency and autonomy*; *Perceived need for cooperation*; *Perception of actual cooperation*; and *Understanding others’ value*. Both total scores and subscale scores can be rated. The internal consistency of the scale as a whole is satisfactory (Cronbach’s alpha = 0.87); however, three of four subscales had an internal consistency below 0.60 [14].

A later version was developed with improved psychometric properties, in particular the test–retest reliability and the subscale structure [15]. The later, adapted scale was developed in a population of Scottish health science students (*n* = 308) and consists of 12 items categorized into three subscales: *Competence and autonomy*; *Perceived need for cooperation*; and *Perception of actual cooperation.* The internal consistency had a Cronbach’s alpha over 0.80 on two of three subscales and higher total scale homogeneity. All subscales approached the 0.60 level for subscale total test–retest reliability. However, the subscale *Perceived need for cooperation,* which only contains two items, gave lower alpha values [15].

The 12-item and three subscale version of IEPS has been previously translated into Swedish and tested in a population containing predominantly medical and nursing students (*n* = 164) [26]. The full scale produced a Cronbach’s alpha coefficient score of α = 0.88 and scores of 0.89, 0.88 and 0.66 respectively for subscales one to three. The factor analyses showed that two items did not significantly load on any factors, and these were therefore deleted. These items were: item 7 “Individuals in my profession trust each other’s professional judgement” (subscale *Competence and autonomy*) and item 8 “Individuals in my profession are extremely competent” (subscale *Perceived need for cooperation*). This loss of two items requires further consideration of convergent and discriminant validity. Also, factor 3, *Perceived need for cooperation,* only contained two items, which does not meet the general rule that requires a minimum of three items per factor [26].

To explore the most appropriate factor structure of the IEPS, a confirmatory factor analysis (CFA) was used in an Australian health profession student and clinical educator population (*n* = 161) [24]. The data were entered into four different IEPS models and demonstrated a lack of model fit for the fit statistics for each of the IEPS models. The lack of model fit argues for further evaluation of construct validity and establishing a version of the IEPS that is sample independent [24]. In a similar study using CFA and comparing the four different IEPS models, the 12-item and three subscale version developed by McFayden et al. appeared to have the closest fit [5]. However, reliability concerns prevailed around the subscale structure, particularly the subscale *Perceived need for cooperation* which had a reliability coefficient below 0.70 [5].

Since several international studies have shown a problematic factor structure of the IEPS using classical test theory, modern test theory has been recommended [24]. Rasch analysis, as part of item response theory (IRT), allows analyses that provide additional information about rating scales, items, and item bias between subgroups [1]. A Rasch analysis of the original 18-item IEPS [14] was performed in an Australian osteopathy student population (*n* = 319) [23]. Using the polytomous Rasch model, they produced a unidimensional 8-item version of the IEPS (IEPS8) that allowed for the calculation of an interval-level total score. By modifying the scale, the item fit residual SD was 0.80, person fit residual 0.98, and the person separation index (PSI) 0.83. Binomial dimensionality testing suggested that the modified IEPS was multidimensional [23]. Despite uncertain psychometric properties, the instrument is widely used to measure students’ perception of IPE and the study population mainly includes nursing and medical students [7, 10, 21, 22].

A recent study developed a new instrument, a *Universal IPE Perceptions Q tool (U-IPEQ)*, containing 40 statements across four domains and comparing it to the two most common IPE evaluation instruments: IEPS and Readiness for Interprofessional Learning scale (RIPLS). The study consisted of twenty students from six disciplines. It showed no statistically significant differences in the IEPS and RIPLS scores before and after the course. However, the U-IPEQ was able to distinguish students’ IPE learning priorities being knowledge and skill. Future research is recommended to further improve the new instrument, including in a larger study population [16]. While a new instrument could improve the evaluation of interprofessional education, the IEPS has been used for almost 35 years, and a substantial amount of data emphasizes the need to continue research on the existing IEPS.

The aim of this study is to further evaluate the psychometric properties of the Swedish version of IEPS in a larger and more varied sample, using Rasch analysis.

## Materials and methods

### Participants and procedure

The participants were all students recruited from eight programmes at the Medical Pharmaceutical Faculty at Uppsala University, Sweden. The programmes included were Speech and Language Pathology, Physiotherapy, Biomedicine, Nursing, Radiography, Medicine, Pharmacology, and Bachelor of Pharmaceutical Science. Students on these programmes have common IPE activities on three occasions during their education: at the beginning of their studies, in the middle of their studies, and towards the end of their studies. Since the study programmes vary in length, the three IPE occasions were defined based on the semester timeline of their respective studies as early (IPE1), middle (IPE2), and late (IPE3). An email was sent to all students before and after they attended an IPE occasion in the autumn semester of 2023. Data collection was conducted between 6th of November 2023 and 24th of January 2024, before and after three IPE occasions, in total on six occasions, labelled “before IPE1”, “after IPE1”, “before IPE2”, “after IPE2”, “before IPE3” and “after IPE3”. Questions included enrolled year level and study programme, age, gender, as well as the questionnaire IEPS. In total, 1176 students completed the questionnaires. One participant was excluded due to missing data. All data included in the study were anonymous. To ensure that all data were from different participants, only data from “before IPE1”, “before IPE2” and “before IPE3” (*n* = 710) were included in the Rasch analysis. The group “unspecified gender” (*n* = 6) was excluded due to having too few respondents for the differential item functioning analysis. The final population was *n* = 704. The majority was female, 78.4% vs. men, 21.6%. The total mean age was 24.7 years, for “before 1” 22.8 years, for “before 2” 24.7 years and for “before 3” 26.6 years. The distribution of participants regarding sex, age, semester, and study programme is shown in Table 1.

**Table 1:** Distribution of participant demographics

	**Rasch analysis, *n* = 704** **n (%)**
Gender	
Female	552 (78.4)
Male	152 (21.6)
Study course	
Before IPE 1	321 (45.6)
Before IPE 2	174 (24.7)
Before IPE 3	211 (29.7)
Study programme	
Pharmacology	104 (14.8)
Biomedicine	52 (7.4)
Physiotherapy	68 (9.7)
Medicine	154 (21.9)
Speech and Language Pathology	15 (2.1)
Bachelor of Pharmaceutical Science	56 (7.9)
Radiography	16 (2.3)
Nursing	239 (33.9)

### Instruments

This study used the Swedish translation of the 12 item, 3 subscale version of the IEPS [15, 26]. The items are divided into the following subscales *Competence and autonomy* (items 1, 3, 5, 7 and 8), *Perceived need for cooperation* (items 4 and 6), and *Perception of actual cooperation* (items 2, 9, 10, 11 and 12). It is a 6-point Likert self-report scale ranging between strongly disagree = 1, moderately disagree = 2, somewhat disagree = 3, somewhat agree = 4, moderately agree = 5 and strongly agree = 6 [15]. The Swedish translation had been performed through a process of forward translations, back-translations, and consensus about discrepancies reached through discussions in the research group, according to recommendations in the literature [6, 26]. The translated Swedish version of the IEPS as developed by Williams (2018) was used in this study and no changes were made to this version. The reliability of the Swedish version showed a Cronbach’s alpha coefficient score of α = 0.88 and scores of 0.89, 0.88 and 0.66 respectively for subscales one to three. The construct validity through factor analyses produced a three-factor solution with eigenvalues > 1, accounting for 77.4% of the total variance [26].

### Statistical method

Data were entered into SPSS (version 28) to organize the data for analysis. Data were then exported from SPSS to R (version 4.4.1) and Rstudio version 2024.04.2 for Rasch analysis. The R packages eRm version 1.0–6, psychotree version 0.16–1, mirt version 1.42 and RISEkbmRasch version 0.2.4.5 were used.

### Rasch analysis

In contrast with classical test theory total score approach, a Rasch analysis more accurately estimates the interaction between a person´s ability and item difficulty. Identifying and improving problematic items allows creating more internally valid and reliable instruments [8]. Psychometric properties and construct validity were analyzed using item response theory, Rasch analysis based on five aspects: dimensionality, rating scale functioning, targeting, invariance, and reliability.

Unidimensionality is a Rasch assumption meaning that items reflect only one main dimension. For unidimensionality to be accepted, the eigenvalue of the unexplained variance of the first contrast should be less than 2 logits [12], which was evaluated by the Principal Component Analysis (PCA). A positive point-biserial correlation indicates that items contribute positively to the total raw score [2]. Also, correlated residuals between item pairs should not be larger than 0.2 above the average of all item-pair residual correlations for local independence.

Item fit was evaluated using the polytomous Rasch model. The data were considered to have a useful fit if mean square standard deviation was between 0.5–1.5 [27]. To be considered useful for the Rasch model, at least 95% of the items should have an *infit* mean square within the range 0.6–1.4, which reflects response patterns for items and whether the item hierarchy is similar for all responders [27]. It is weighted by its statistical information (model variance) and is more sensitive to unexpected patterns of observations by persons regarding items that are roughly targeted on them. The *outfit* range, 0.6–1.4, of at least 95% of the items is more sensitive to the unexpected observations by persons regarding items that are relatively very easy or very hard for them [27].

Rating scale functioning recommends that each response category should include at least 10 observations, the outfit mean square (MnSq) should be below 2.0, average measures and step difficulty for each category should increase (higher logit value), and categories should be ordered as intended, with an acceptable distance between adjacent categories. This was measured using a partial credit model (PCM) with a recommended distance of 1.4 to 5 logits and item probability functions [19].

### Targeting between item difficulty and participant ability

Targeting of items compared to participants was reviewed by visualizing the distribution of person locations on the same scale as the distribution of item thresholds. This was performed by using an item-hierarchy map that displays the location of person abilities and item difficulties, respectively, along the same latent dimension. The Wright map, also known as the item-person map, was used in order to examine the difficulty of the items on the same measurement scale as the respondent’s latent trait, i.e. the ability of the students. An item characteristic curve (ICC) for each item shows the probability of a correct response as a function of the ability of the respondent by using two scores, the item difficulty and the total score on the test. The distance between item and person means that between ceiling and floor effects were evaluated.

Invariance was evaluated for item bias using Differential item functioning (DIF), to differentiate if any demographic variables (age above and below 25 years and gender) significantly influenced the way an item was responded to [3]. To identify any statistical significance between groups, the following criteria applied: (1) DIF size (difference between item measurements) large enough to have substantial consequences between groups of > 0.5 logits and (2) a statistical significance level (*p*-value) < 0.05 [12].

### Reliability and separation of persons and items

The reliability of IEPS was measured as internal consistency using Cronbach’s alpha, with satisfactory α values defined as 0.7 ≤ α ≤ 0.9 [20]. Person separation index is an additional reliability index. Person separation index (PSI) evaluates the ability of the items to measure different levels of latent construct. A low PSI (< 2, person reliability < 0.8) may indicate that the instrument is not sensitive enough to distinguish between persons based on their ability [13].

## Results

### Unidimensionality

Concerning dimensionality of the whole instrument, the PCA of residuals showed an eigenvalue higher than recommended (2.09) for the main dimension. This implies that IEPS is multidimensional.

Regarding overall item fit, 11 items out of 12 were in the recommended range for the infit mean square and 10 items out of 12 were in the recommended range for the outfit mean square. Item 6 (Individuals in my profession must depend upon the work of people in other professions) indicated a misfit in both infit (mean square 1.631 logits) and outfit (mean square 3.134 logits). Item 4 (Individuals in my profession need to cooperate with other professions) indicated a misfit in outfit (mean square 1.480 logits), see Table 2.

**Table 2:** Fit statistics for the Swedish version of 12-item IEPS, *infit* and *outfit* mean square reflects response patterns for items and unexpected observations by persons on items. After recalculation based on thresholds from 1000 simulated data sets values outside the proposed range is shown as red numbers

Item	Infit MSQ	Infit tresholds	Outfit MSQ	Outfit tresholds	Infit diff	Outfit diff
1	1.08	[0.884, 1.133]	1.123	[0.836, 1.185]	No misfit	No misfit
3	0.922	[0.886, 1.104]	0.926	[0.859, 1.131]	No misfit	No misfit
5	0.973	[0.891, 1.114]	0.993	[0.883, 1.156]	No misfit	No misfit
7	0.859	[0.884, 1.116]	0.853	[0.871, 1.141]	0.025	0.018
8	0.95	[0.896, 1.116]	0.957	[0.85, 1.219]	No misfit	No misfit
4	1.154	[0.897, 1.123]	1.462	[0.876, 1.142]	0.031	0.32
6	1.685	[0.832, 1.148]	3.234	[0.73, 1.471]	0.537	1.763
2	0.903	[0.873, 1.132]	0.853	[0.864, 1.137]	No misfit	0.011
9	0.877	[0.857, 1.148]	0.915	[0.761, 1.361]	No misfit	No misfit
10	0.891	[0.881, 1.127]	0.873	[0.876, 1.141]	No misfit	0.003
11	0.965	0.864, 1.113[]	0.977	[0.859, 1.121]	No misfit	No misfit
12	0.772	[0.879, 1.137]	0.768	[0.85, 1.193]	0.107	0.082

Item 4 and item 6 showed a residual correlation of 0.44, indicating that the item does not have local independence. Item-pair correlations of item residuals, categorized in subscales, are shown in Table 3.

**Table 3:**
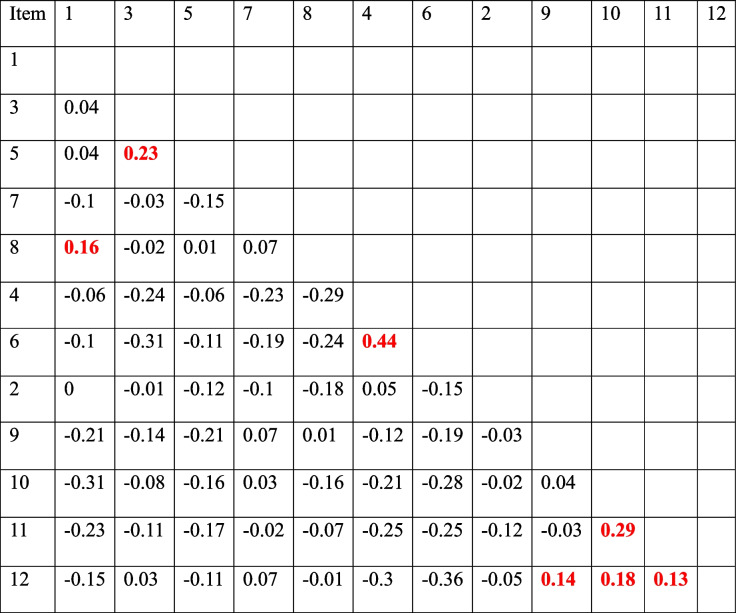
Item-pair correlations of item residuals, categorized in subscales. Highlights in red show correlations above 0.124, which is 0.2 above the average item-pair correlation (−0.076)

**Fig. 1:**
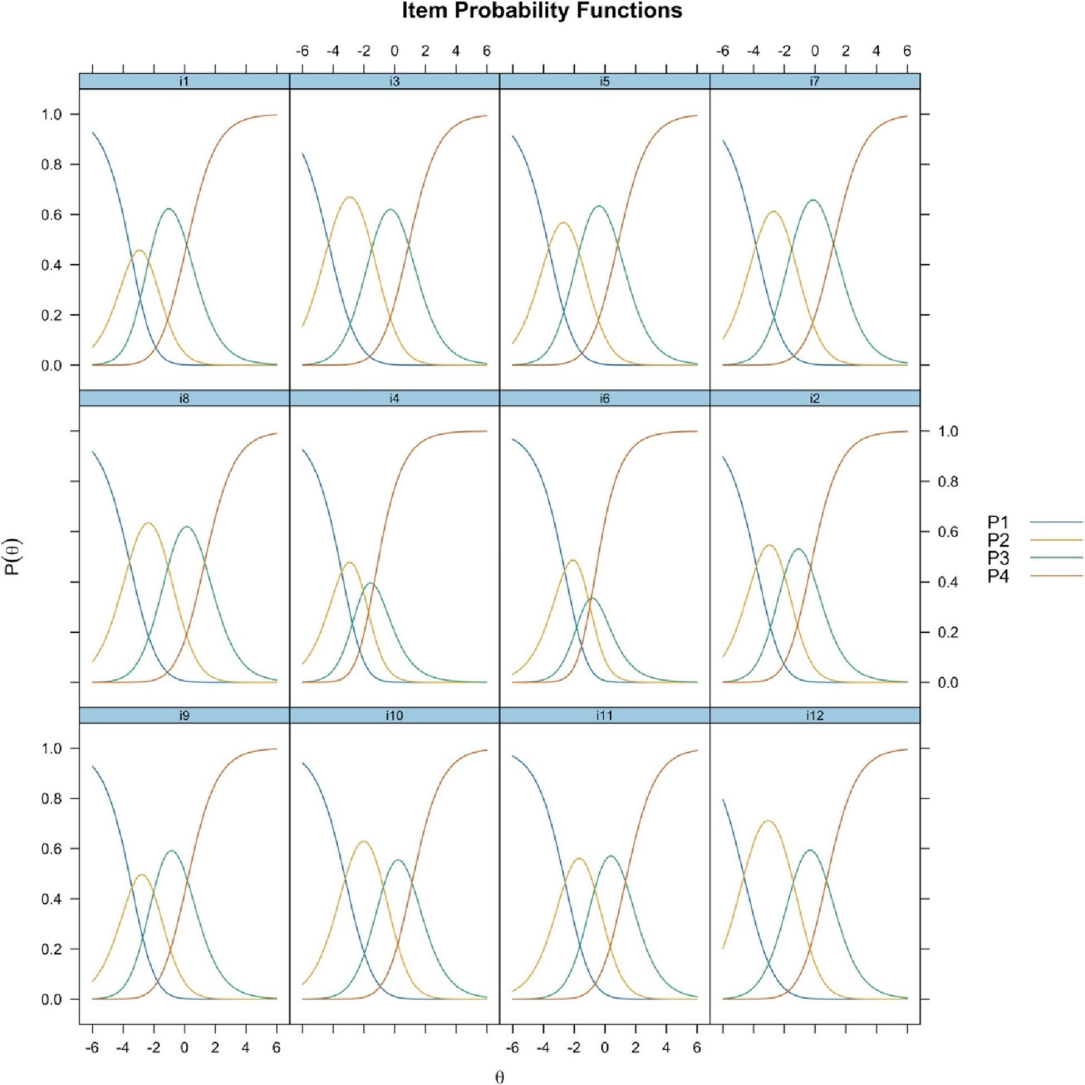
Rating scale category structure, item probability functions, visualizing the distribution of person locations on the same scale as the distribution of item thresholds, presented according to the subscale items. Categories P1-P4 reflect assumed increased level of agreement

Item probability functions showed disordered categories of thresholds 1 and 2 for item 6 and threshold 1 for item 4, indicating that analyst-assigned category order does not accord with the latent variable. All other items showed clearly advancing and ordered thresholds, see Fig. 1.

### Rating scale functioning

**Fig. 2:**
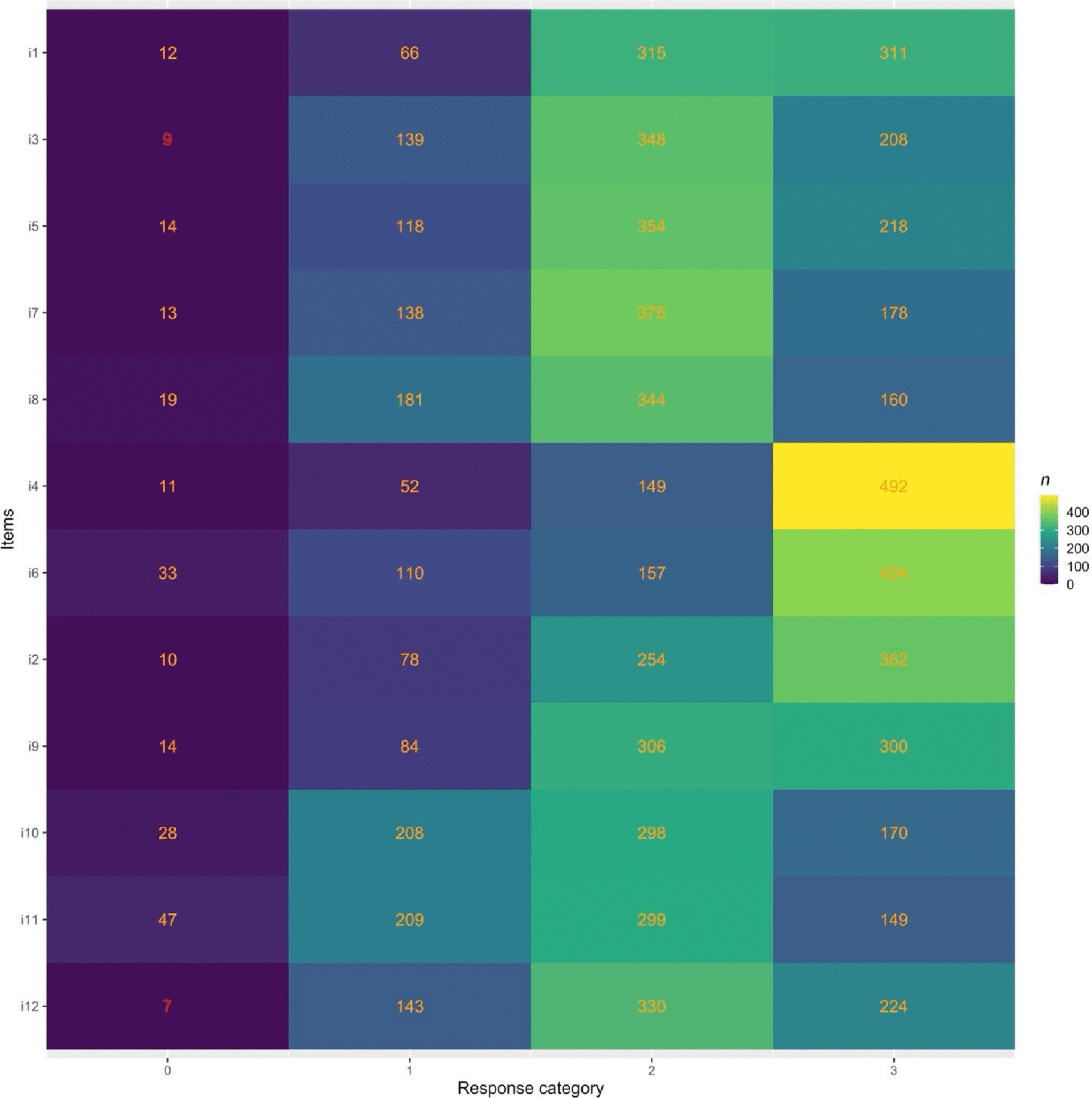
Response categories, with polytomous (ordered) response categories for disagreement combined into response category 0 (left column). Category 1 shows number of responses for somewhat agree = 4, category 2 shows number of responses for moderately agree = 5 and category 3 shows number of responses for strongly agree = 6

Initial analysis of the six item response categories showed responses below 10 observations. The IEPS was, therefore, not suitable for a polytomous Rasch model on initial analysis. In order to improve step calibration and the stability of the analysis, and reaching 10 observations in each category [12], categories 1, 2 and 3 were combined into one response category. Despite combining these categories, two items (item 3 and item 12) still had below 10 observations, 9 and 7 observations respectively, see Fig. 2.

Partial Credit Model (PCM) showed an increase in logit value for each item. However, item 6 did not meet the recommended distance of 1.4 to 5 logits between thresholds 1 and 2, 0.07 and 0.15 logits respectively, see Table 4.

**Table 4:** Partial credit model (PCM), average measures and step difficulty for each category should increase (higher logit value) and categories should be ordered as intended, with an acceptable distance between adjacent categories (1.4 to 5 logits)

	**Thresholds**		
Item	**1**	**2**	**3**
1	−0.98	1.41	2.44
2	−0.53	1.06	2.98
3	−1.75	0.77	2.79
4	0.51	1.08	2.59
5	−1.69	0.77	2.79
6	**0.07**	**0.15**	1.68
7	−2.08	0.64	2.98
8	−2.19	0.19	2.62
9	−1.02	1.11	2.53
10	−1.20	−0.12	2.31
11	−2.23	−0.17	1.67
12	−1.59	0.53	3.98

### Targeting between item difficulty and participant ability

**Fig. 3:**
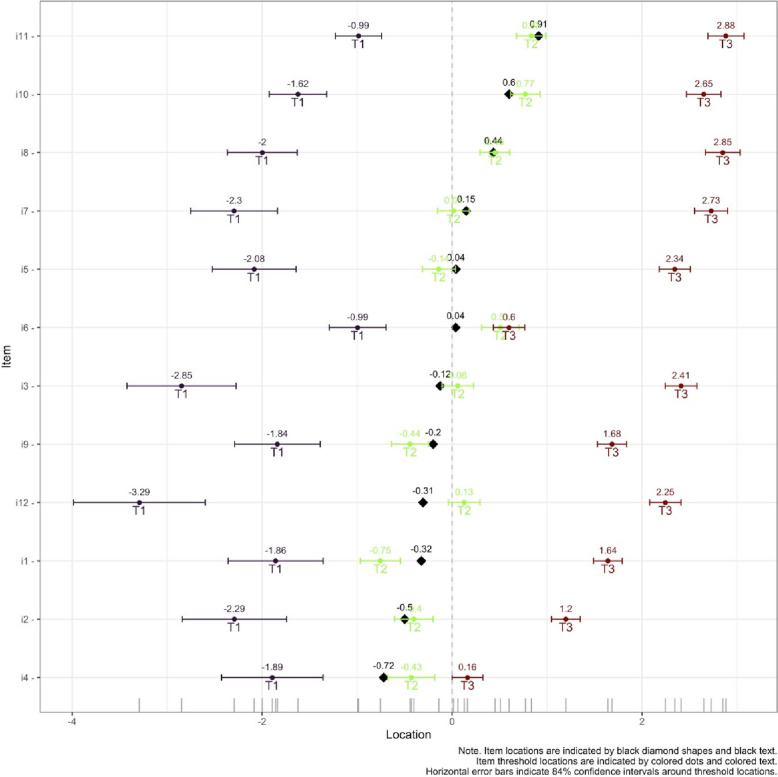
Item-hierarchy map for the Swedish IEPS in health care students. The person ability parameter is located on the left (low) to right (high). Item locations are indicated by black diamond shapes and black text. Item threshold locations are indicated by coloured dots and coloured text. Horizontal error bars indicate 84% confidence intervals around threshold locations

An item-hierarchy map showed disordered thresholds for items 4 and 6, see Fig. 3.

**Fig. 4:**
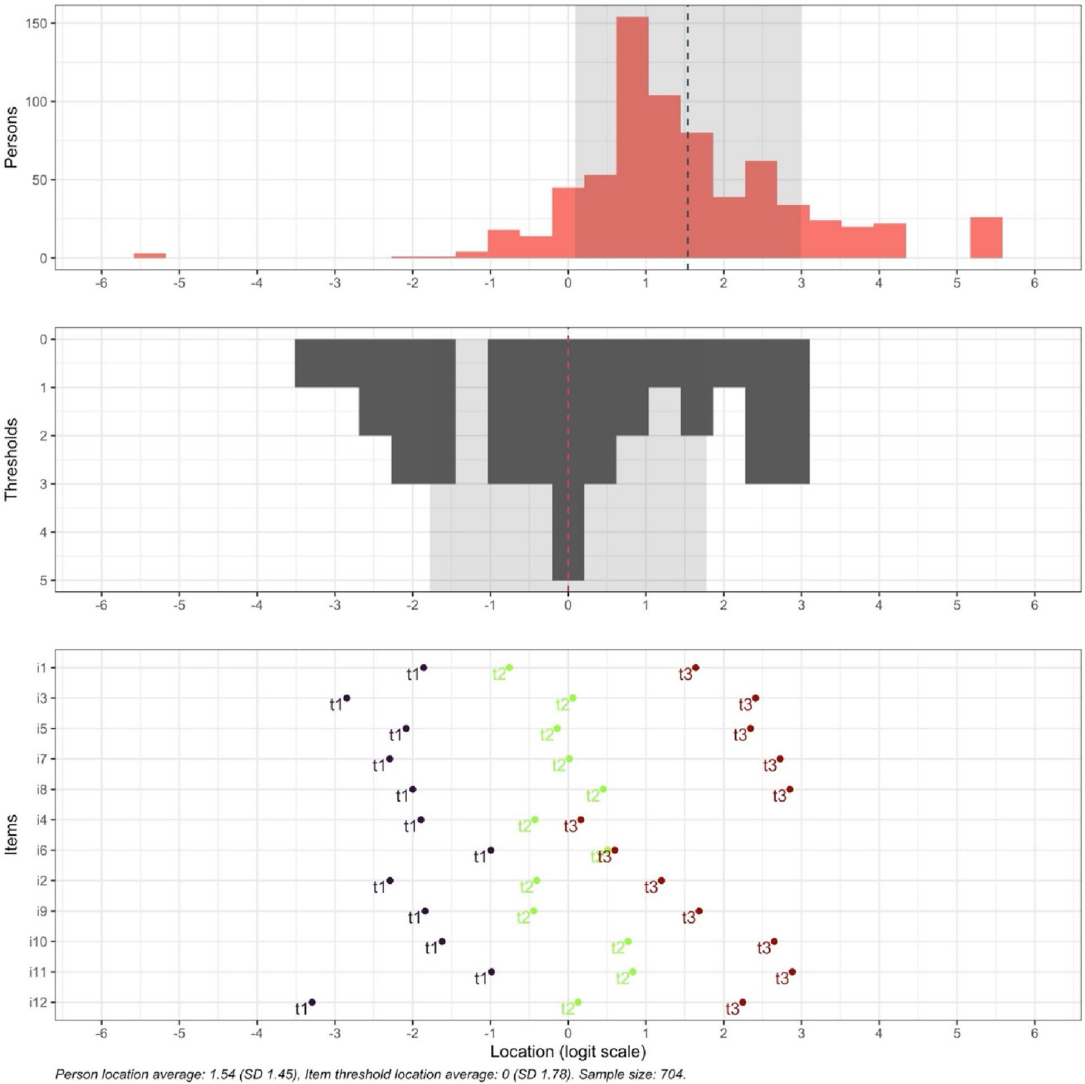
Wright map, which visualizes the comparison between person ability and item difficulty. Respondent latent trait (person’s ability) shown on the top and item difficulty (thresholds) shown below. The logit scale below shows person ability and item difficulty on the same interval scale, where a negative value represents lower ability or easier item and a positive value represent higher ability or more difficult item

The distribution of respondent latent trait scores in the Wright item-person distribution map was not evenly distributed. Item difficulty analysis showed that items 12 and 6 were difficult items, items 9 and 5 were medium difficult items, and the remaining items were easy. Overall, most participants were well-targeted by items, but the sample did show some ceiling/floor effects, see Fig. 4.

**Fig. 5:**
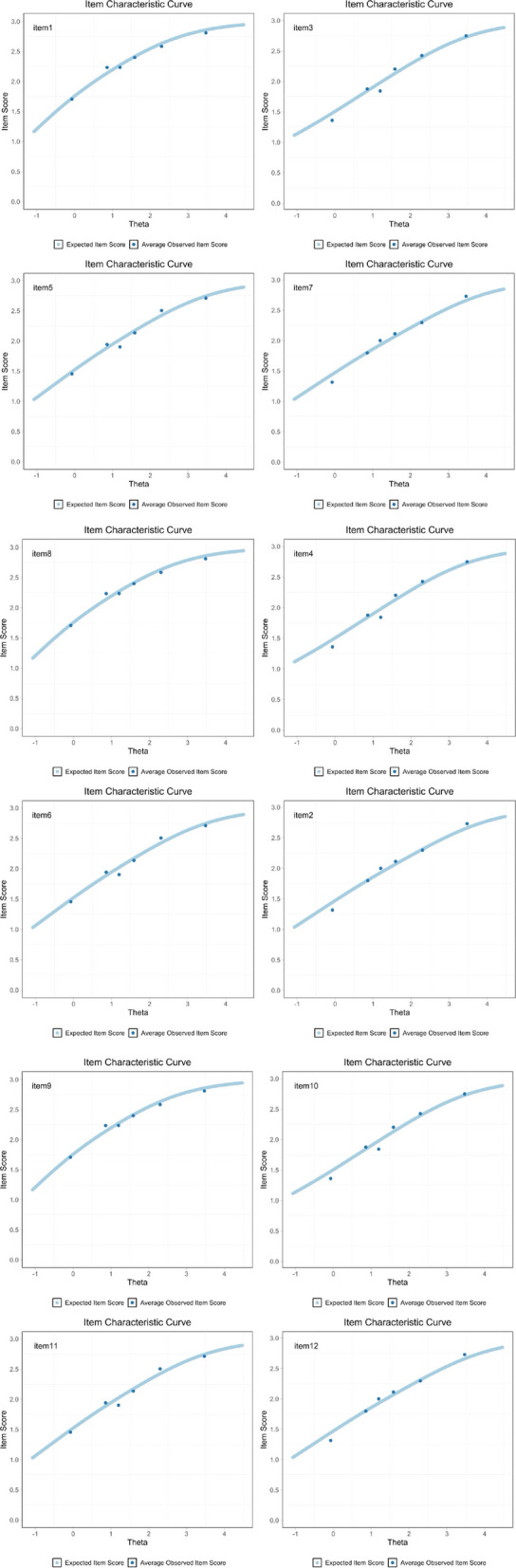
Item curve characteristics showing the expected item score as a light blue curve and average observed score as dark blue dots. It shows how the probability of choosing a response on Y-axis (e.g. getting an item correct or selecting a higher category) and on the X-axis (Theta) changes with a person´s ability level

The item characteristic curve showed that most items could discriminate between those participants who perform high versus low on the total score. However, item 6 was shown to be a poor discriminator, with the dots not fitting on the ascending curve, see Fig. 5.

### Invariance (Differential item functioning (DIF))

When all Rasch criteria were met, by deleting item 6 and item 4, the DIF analysis showed no significant difference in item bias based on gender or age.

### Reliability and separation of persons and items

Internal consistency for IEPS using Cronbach’s alpha was 0.83, indicating satisfactory internal consistency [9]. Person separation index was 0.84 (again after meeting Rasch criteria and deleting item 6 and item 4).

## Discussion

This study explored the psychometric properties of the Swedish version of IEPS using Rasch analysis. The scale was suboptimal in aspects of the response categories and with regard to the second subscale, *Perceived need for cooperation*, containing item 4 and item 6.

Rating scale analysis indicated problems with the response categories. Concerning the distribution of responses, very few (less than 10) were noted in the response categories for disagreement (categories somewhat disagree = 3, moderately disagree = 2, and strongly disagree = 1) and these were combined into one response category for disagreement. Despite combining these categories, two items (item 3 and item 12) had below 10 observations, 9 and 7 observations respectively. The ceiling/floor effects shown in the Wright map are likely due to the distribution of response categories. Problems with the response categories have not been discussed in previous research, neither in the original English version nor the Swedish version [15, 26]. Therefore, future research needs to pay attention to the response categories, possibly rephrasing them or minimizing the number of response options.

The second subscale, *Perceived need for cooperation,* contains item 4 (Individuals in my profession need to cooperate with other professions) and item 6 (Individuals in my profession must depend upon the work of people in other professions). This subscale has been problematic with regards to classical test theory, for both reliability and factor analysis [15, 24, 26]. Also using Rasch analysis, this subscale was troublesome. Item fit statistics showed a residual correlation between items 4 and 6, indicating that these items do not have local independence. Violations of item local independence can lead to inflated estimates of reliability and problems with construct validity [4]. Both items had outfit values above the recommended value and item 6 also had an infit value above recommendations. Item 6, and to some extent item 4, were disordered in the item probability functions analysis. The ability of the students, therefore, does not reflect on item probability for these items. Both items 4 and 6 showed disordered thresholds, meaning that the ability (latent variable) does not predict the response. Item 6 was also shown on the item characteristic curve to not be able to discriminate between low and high ability respondents.

Previous research has shown that no model of IEPS has consistently demonstrated fit statistics at or above recommended levels, which suggests measurement invariance or misfit due to chance [24]. Dimensionality testing did not support unidimensionality of the IEPS, but showed that the instrument is multidimensional. This is in line with previous research where, to achieve unidimensionality, Vaughan [23] used Rasch analysis of the 18-item version of the instrument and kept only eight items. Among the removed items were items 6 and 4. Item 6 was removed due to misfit in item statistics, with poor item fit residuals, which is in accordance with this study, and item 4 was removed due to the DIF for year level [23].

Consequently, this subscale requires revision. In order to improve reliability and validity, item 6 should be removed from the scale. This, however, will leave only item 4 as one factor. Future research should perform a factor analysis through classical test theory to evaluate if item 4 might load onto another factor or if it should also be removed from the scale, leaving the scale as a two-factor scale but with potentially improved psychometric properties. Practical implications of removing the second subscale mean that the IEPS no longer measures the full construct it was designed to assess. This may decrease construct and content validity, can lower overall reliability and may create issues with cross-study comparability to previous research. However, the theoretical implications may improve the psychometric properties of the scale.

Overall, the results showed that the total scale had satisfactory internal consistency, consistent with previous research [15, 24, 26]. Person separation index showed that the instrument overall can separate between participants ability, when item 6 and item 4 are deleted. Finally, the DIF analysis showed no significant difference in item bias based on gender or age.

### Limitations

Considering that the previous study using classical test theory also showed items 7 and 8 failing to load on any factor [26], which was not shown in this study, this inconsistency emphasizes the need for more research on the Swedish translation.

Although a sample size of 704 is deemed sufficient for a Rasch analysis, female nursing students were overrepresented in the participants. All data were collected anonymously, which means test–retest reliability could not be tested. Also, the participants were self-recruited, which might have created a self-selection bias, either positive or negative. Secondly, the Swedish translation of the IEPS has only been tested on one previous population where mean scores were significantly higher than in this study [26]. This could be explained by differences in populations, since the original Swedish study had fewer participants and less study programmes represented. A major limitation is the lack of a replication of the findings in an independent sample. The revised version therefore needs to be further evaluated. Also to be considered are the cultural and linguistic discrepancies that might have changed over time, since the original scale was constructed almost 35 years ago. Future cohorts using the Swedish translated instrument would enable further comparisons.

## Conclusion

Using modern test theory, Rasch analysis, this study shows that the Swedish IEPS in its current form is suboptimal. This study shows that a re-evaluation of the IEPS should be carried out, especially regarding the response categories and the second subscale, *Perceived need for cooperation*. The distribution of responses, less than 10 observations were noted in the response categories for disagreement. Therefore, future research needs to evaluate rephrasing the response categories or minimizing the number of response options. Additionally, the second subscale should be revised. The removal of item 6 and possibly item 4 may improve the psychometric properties of the scale, this also removes the possibility to evaluate the perceived need for cooperation.This study contributes to the further development of the IEPS regarding psychometric properties and creating a more reliable instrument to assess students’ perception of IPE and consequently interprofessional competency.

In conclusion, response categories and the second subscale need revision to improve the evaluation of interprofessional education using the IEPS.

## Data Availability

The data that support the findings of this study are available on reasonable request from the corresponding author.

## Abbreviations

IEPS: Interdisciplinary education perception scale
IPE: Interprofessional education
IPL: Interprofessional learning
CFA: Confirmatory factor analysis
IRT: Item response theory
PSI: Person separation index
MnSq: Mean square
PCM: Partial credit model
PCA: Principal component analysis
ICC: Item characteristic curve
DIF: Differential item functioning
